# Évaluation des pratiques et connaissances en radioprotection des cardiologues en Tunisie

**DOI:** 10.11604/pamj.2021.38.300.24254

**Published:** 2021-03-23

**Authors:** Rania Hammami, Abdelhamid Ben Jmaa, Amine Bahloul, Selma Charfeddine, Tarek Ellouze, Souad Mallek, Imtinene Ben Mrad, Leila Abid, Samir Kammoun, Jihen Jdidi

**Affiliations:** 1 Service de Cardiologie, Hôpital Hedi Chaker, Sfax, Tunisie,; 2 Unité de Recherche UR 17ES37, Faculté de Médecine de Sfax, Université de Sfax, Sfax, Tunisie,; 3 Service de Cardiologie Habib Thameur, Tunis, Tunisie,; 4 Service de Médecine Préventive, Hôpital Hedi Chaker, Sfax, Tunisie

**Keywords:** Rayonnements ionisants, connaissances, questionnaire, cardiologie interventionnelle, Ionizing radiations, knowledge, survey, interventional cardiology

## Abstract

**Introduction:**

les procédures de cardiologie interventionnelle sont devenues complexes et chronophages avec un sur-risque d´exposition aux rayonnements ionisants. L’objectif de notre étude était d’évaluer le niveau des connaissances et des pratiques en radioprotection des cardiologues Tunisiens exposés au rayon X.

**Méthodes:**

notre étude est descriptive analytique réalisée en octobre 2019, organisée sous forme de questionnaire anonyme des connaissances et pratiques en radioprotection et envoyé à tous les Cardiologues Tunisiens exposés aux rayons X.

**Résultats:**

parmi 126 cardiologues exposés aux rayons X et ayant reçu le questionnaire, 58 médecins ont répondu au questionnaire (48%), avec une prédominance masculine (72%, n=42). Trente-huit médecins (65%) exerçaient dans le secteur public. L´expérience professionnelle était en moyenne de 12,02 ans (ET 6,88 ans). La moitié des médecins avaient un score de connaissances inférieur à 50%. La moyenne du score des pratiques était de 43,83 (ET 13,95%). Le port du tablier en plomb, de la cache thyroïde, du dosimètre, des lunettes en plomb, du calot en plomb était respectivement de 100% (n=58), 86,2% (n=50), 30,7% (n=18), 12,1% (n=7) et 1,7% (n=1). Il n´y avait pas de corrélation entre les scores et l´âge du médecin ainsi que la durée de l´expérience professionnelle. Les scores de connaissances ne différaient pas statistiquement entre les 2 sexes (p=0,06) ni entre le secteur public et le secteur privé (p=0,9). Le score de pratique était significativement plus élevé chez les hommes (0.007) et les cardiologues interventionnels comparés aux rythmologues et cardiopédiatres (p<0.001).

**Conclusion:**

le niveau des connaissances et des pratiques des cardiologues Tunisiens en radioprotection est globalement insuffisant. Ce qui interpelle les autorités sanitaires à organiser un plan de formation régulier pour cette population.

## Abstract (en)

**Introduction:**

interventional cardiology procedures have become complex and expensive in time with a higher risk of exposure to ionizing radiations. The purpose of our study was to assess radiation protection knowledge and practices among Tunisian cardiologists exposed to X-rays.

**Methods:**

we conducted a descriptive, analytical study in October 2019. An anonymous survey on the knowledge and practices regarding radiation protection was sent to all Tunisian cardiologists exposed to X-rays.

**Results:**

among 126 cardiologists exposed to X-rays and having received the survey, 58 physicians responded to the survey (48%), with a male predominance (72%, n=42). Thirty-eight physicians (65%) were public sector workers. Average years of working experience were 12.02 years (SD 6.88 years). Half of doctors had a knowledge score of less than 50%. The average practice score was 43.83 (SD 13.95%). Wearing a lead apron, thyroid shield, dosimeter, lead glasses, lead cap accounted for 100% (n=58), 86.2% (n=50), 30.7% (n=18), 12.1% (n=7) and 1.7% (n=1) respectively. There was no correlation between scores and doctor’s age as well as the length of working experience. There was no statistical differences between knowledge scores of males and females (p=0.06) or between public sector and private sector (p=0.9). Practice score was significantly higher among men (0.007) and interventional cardiologists compared to rhythmologists and pediatric cardiologists (p<0.001).

**Conclusion:**

the level of knowledge and practices among Tunisian cardiologists regarding radiation protection is generally insufficient. Then, health authorities should implement regular training programs.

## Introduction

Les innovations techniques au cours des dernières décennies ont rendu possible la réalisation de procédures de cardiologie interventionnelle complexes relevant auparavant d’une gestion médicale ou d’une prise en charge chirurgicale. Il en résulte une exposition plus importante des patients et des praticiens aux rayonnements ionisants (RI) [1], donc une nécessité de bonne connaissance de la dose des RI reçue par les opérateurs, des facteurs influençant la dose et des possibilités pour la réduire. L’objectif de notre étude était d’évaluer le niveau de connaissance et des pratiques des cardiologues interventionnels en Tunisie.

## Méthodes

Notre étude était descriptive analytique rétrospective réalisée en octobre 2019, ayant concerné des cardiologues interventionnels exerçant en Tunisie. Nous avons utilisé un questionnaire anonyme préétabli par un cardiologue interventionnel en se référant aux données de la littérature.

**Population de l´étude:** notre population cible était constituée de tous les cardiologues exerçant en Tunisie et exposés professionnellement aux rayons X.

**Le questionnaire:** pour évaluer les connaissances de l´exposition aux rayons X et les pratiques en matière de radioprotection, un questionnaire a été préétabli en se référant aux données de la littérature par un médecin hospitalo-universitaire cardiologue ayant reçu une formation approfondie en radioprotection. Le questionnaire d´évaluation, anonyme, a été envoyé par internet (formulaire Googleform anonyme) aux médecins avec une explication courte des objectifs de l´enquête et remis par internet. Ce questionnaire comportait 3 rubriques et au total 37 questions. La 1^e^ rubrique a porté sur les caractéristiques socioprofessionnelles et personnelles : le sexe, l´âge, la sous-spécialité, la durée de l´expérience professionnelle, l´obtention ou non d´une formation en radioprotection, l´antécédent de stérilité ou de fausse couche en rapport avec le travail en salle de cathétérisme, l´antécédent de malformation physique ou mentale auprès de la descendance et le retentissement des grossesses ou des projets de grossesse sur le parcours professionnel.

La 2^e^ rubrique comportait des questions concernant les connaissances en radioprotection : les caractéristiques des rayons X, les valeurs limites annuelles d´exposition à ne pas dépasser, les moyens de surveillance de l´exposition aux rayons X, la perception du risque des RI sur la santé, l´aptitude de la femme de travailler en salle au cours de la grossesse La 3^e^ rubrique portait sur les pratiques en matière de radioprotection. Pour l´évaluation des connaissances, nous avons calculé pour chaque participant un score attribuant 1 point pour une bonne réponse et 0 point pour une réponse fausse. Ce score a été établi sur 8 questions. Le résultat final a été exprimé en pourcentage. A noter que les réponses manquantes ont été considérées comme fausses. De même, l´évaluation des pratiques s’est basée sur un score attribuant 2 points pour ceux qui adhèrent toujours aux bonnes pratiques, 1 point pour ceux qui appliquent irrégulièrement les bonnes pratiques et 0 point pour ceux qui ne suivent pas les règles de radioprotection. Nous avons calculé ainsi un score global de pratique établi sur 14 questions, exprimé en pourcentage.

**Analyse statistique:** les données ont été saisies par le logiciel EXCEL et analysées au moyen du logiciel SPSS version 19. Les variables quantitatives ont été exprimées en moyenne ± écart type ou en médiane selon que la distribution était gaussienne ou non. Les variables qualitatives ont été exprimées en fréquences simples et en pourcentage. Nous avons réalisé une étude analytique testant les variables une à une afin d´identifier les facteurs pouvant avoir un impact sur les connaissances et les pratiques des médecins. Cette analyse s’est basée sur les tests paramétriques et non paramétriques selon la distribution des variables quantitatives. Nous avons utilisé le test de Mann-Whitney et le test T de Student pour comparer les moyennes sur les séries indépendantes. Pour la corrélation entre 2 variables quantitatives, on a utilisé la corrélation de Spearman et de Pearson. Le seuil de significativité a été fixé à 0.05.

## Résultats

### Etude descriptive

**Caractéristiques socioprofessionnelles de notre population:** dans notre pays, il existe 447 spécialistes en cardiologie, dont 318 médecins qui pratiquent dans le secteur privé (71%) et 109 de sexe féminin (24.3%). Tous les cardiologues Tunisiens exposés aux rayons X (n=126) avaient reçu le questionnaire par mail, 58 ont répondu soit un taux de réponse au questionnaire qui était à 46% (58/126). La majorité de la population était de sexe masculin, soit 42 hommes (72%). L’âge moyen était de 41,87 (ET 6,06 ans). Trente-huit médecins (65%) exerçaient dans le secteur public. L´expérience professionnelle était en moyenne de 12,02 (ET 6,88 ans). La cardiologie interventionnelle était la sous-spécialité la plus représentée 79% (n= de tous les médecins suivis par la rythmologie (17%) et la cardiologie pédiatrique (4%). Parmi les répondants, 53,4% ont eu une formation en radioprotection avant d´exercer en salle de cathétérisme. Parmi eux, 4 médecins (12,9%) ont eu une formation en Tunisie. Dans notre population, 6 enquêtés (10,34%) avaient rapporté une stérilité en rapport avec leur travail en salle de cathétérisme et 2 médecins (3,44%) ont rapporté des antécédents de malformations physiques ou mentales dans leur descendance. Parmi les 16 femmes, 43,75% avaient des antécédents de fausse couche après avoir travaillé en salle de cathétérisme. Concernant les femmes ayant eu des grossesses pendant l´exercice en salle de cathétérisme (n=14), 57,14% ont arrêté de travailler lors de la grossesse. Parmi celles qui ont continué de travailler en salle au cours de la grossesse, 66,66% ne savaient pas qu´elles étaient enceintes. D´autre part, 62,5% des femmes (n=10) ont rapporté que les grossesses ou les projets de grossesses ont retenti sur leur parcours professionnel. Aucun médecin n´a été opéré sur la thyroïde.

**Etude des connaissances:** la médiane du score des connaissances était de 55,55% (11, 11-88, 89). Concernant l´étude des connaissances, le pourcentage de réponses correctes par question a varié entre 24,1% et 87,7%. Sur 8 questions, 4 questions seulement ont eu un taux de réponses correctes qui dépassent les 50% (Tableau 1).

**Tableau 1:** réponses correctes selon la question

Questions	Réponses correctes	
	Nombre	Pourcentage en %
La distance minimale à partir de laquelle les rayons X deviennent sans danger	**14**	**24,1**
Port du dosimètre	**21**	**36,2**
Influence des pratiques sur l´exposition des opérateurs au fils du temps	**51**	**87,7**
Le risque de cataracte radio induite après une exposition au rayon X de plus de 10 ans	**35**	**60,3**
le risque du cancer du cerveau après une exposition au rayon X de plus de 10 ans	**32**	**55,2**
la dose limite annuelle à ne pas dépasser pour les personnes exposées au rayon X	**30**	**51,7**
Possibilité du travail de la femme enceinte en salle de cathétérisme	**26**	**44,8**
Aptitude du médecin atteint de cataracte pour travailler en salle de cathétérisme	**16**	**27,6**

**Etude des pratiques:** la moyenne du score des pratiques était de 43,83 (ET 13,95) (Tableau 1). Les pratiques concernant les moyens de radioprotection et la signalisation des rayons X: les 2/3 des opérateurs n´ont jamais marqué les paramètres d´exposition aux rayons X dans leurs comptes rendus. Dans notre étude, l´adhérence au port du tablier en plomb, de la cache thyroïde, des lunettes en plomb, du calot en plomb était respectivement de 100% (n=58), 86,2% (n=50), 12,1% (n=7) et 1,7% (n=1) (Tableau 2); les pratiques concernant les moyens de surveillance médicale et dosimétrique de l´exposition aux rayons X: trente-cinq médecins soit 60,3% n´ont jamais porté de dosimètre en raison de la non disponibilité chez 31 médecins, la perception de la non fiabilité des données dosimétriques chez 3 médecins et l´oubli chez un seul médecin. Uniquement 15,5% des médecins portaient toujours le dosimètre; Les pratiques concernant les principaux intervenants en radioprotection: parmi notre population, 45 médecins (77,5%) n´ont jamais consulté en médecine du travail au cours de leur activité et 13 médecins (22,4%) ont consulté uniquement une seule fois. Vu le risque de cataracte radioinduite, uniquement 11 médecins (19%) consultent régulièrement un ophtalmologue. Parmi ceux qui n´ont pas consulté, 68% connaissaient le risque mais n´avaient pas assez du temps pour consulter.

**Tableau 2:** adhérence des enquêtés aux bonnes pratiques de radioprotection

Pratiques	Jamais (%)	Parfois (%)	Toujours (%)
Port du tablier en plomb	**0(0)**	**0(0)**	**58(100)**
Port du cache thyroïde	**1(1,7)**	**7(12,1)**	**(86,2)**
Port du calot en plomb	**53(91,4)**	**4(6,9)**	**1(1,7)**
Port des lunettes en plomb	**35(60,3)**	**16(27,6)**	**7(12,1)**
Utilisation du filtre lors de l´acquisition des images	**10(17,2)**	**25(43,1)**	**20( 34,5)**
Utilisation de la collimation	**12(20,7)**	**32(55,2)**	**14(24,1)**
Utilisation de l´option sauvegarde Scopie	**11(19)**	**38(65,5)**	**9(15,5)**
Utilisation de la barrière vitrée de protection lors des procédures	**4(6,9)**	**2(3,4)**	**12(20,7)**
Utilisation adaptée de l´agrandissement de l´image en fonction du temps de la procédure	**18(31)**	**-**	**37(63,8)**
Vérification du nombre d´images/seconde utilisé lors des procédures	**23 (39,7)**	**-**	**35(60,3)**

### Etude analytique

**Facteurs corrélés aux bonnes connaissances:** l´étude des facteurs associés aux bonnes connaissances n´a pas montré de différence statistiquement significative entre les 2 sexes, le secteur public et privé et les différentes sous spécialités (Tableau 3). Nous n´avons pas également trouvé une corrélation significative entre le score des connaissances et l´âge ou la durée de l´expérience professionnelle (Tableau 3).

**Tableau 3:** corrélation des scores d´évaluation avec l´âge et l´expérience professionnelle

	Score de connaissances		Score de pratiques	
	Coefficient de Spearman	P	Coefficient de Spearman	P
Âge	-0,04	0,74	0,215	0,106
Expérience professionnelle	0,02	0,85	0,105	0,43

**Facteurs associés aux pratiques:** l´étude des facteurs associés aux bonnes pratiques a montré une différence significative entre les hommes et les femmes et entre les différentes sous spécialités (Tableau 4).

**Tableau 4:** étude du score de connaissance en fonction des paramètres qualitatifs

Paramètre qualitatif		Score de connaissance % (ET)	p	Score de pratiques (%)	p
Sexe	Homme(n=42)	49,47(16,49)	0,06	46,15# [15,38-76,92]	0,007
	Femme(n=16)	59,02(19,75)		32,69# [23,08-53,85]	
Secteur	Public(n=38)	55,55 # [11,11-88,89]	0,9	43,52(14,19)	0,84
	Privé(n=20)	55,55# [33,33-77,78]		44,42(13,8)	
Sous spécialité	Interventionnelle (n=46)	50,41(17,05)	0,26*	47,84(13,46)	<0,001*
	Rythmologie (n=10)	60(21,72)	0,12*	32,30(11,05)	0,003*
	Cardiologie pédiatrique (n=2)	50,79(15,52)	0,83*	36,81(8,26)	0,15*

* p : résultat de la comparaison du groupe correspondant avec les autres sous spécialités # : médiane

**Corrélation entre les scores des connaissances et des pratiques:** dans notre étude, nous n´avons pas trouvé de corrélation statistiquement significative entre le score des connaissances et des pratiques (P=0,523).

## Discussion

La radioprotection est rendue obligatoire en Tunisie conformément à la loi relative aux rayonnements ionisants (loi 81-51 du 18 juin 1981; République Tunisienne, 1986). La radioprotection des patients, des travailleurs et des membres du public repose sur les principes de justification des pratiques, d´optimisation des expositions et de limitation des doses reçues par les travailleurs [2]. La cardiologie est considérée actuellement comme le domaine ou les travailleurs sont le plus exposés aux Rayons X. En Amérique, les cardiologues sont impliqués dans environ 40% d’irradiation médicale en dehors de la radiothérapie [1,3]. Les cardiologues interventionnels ont une exposition personnelle annuelle d’environ 5msv, trois fois plus élevé que les radiologues et les médecins nucléaires. Ils ont un sur-risque de cancer attribuable à la vie professionnelle de l’ordre de 1 sur 100 [4]. Ceci pourrait expliquer leur meilleure adhérence aux bonnes pratiques même par rapport aux autres sous-spécialités dans notre étude. Aussi, la radioprotection des travailleurs rend obligatoire le respect des limites des doses de RI à ne pas dépasser chez les travailleurs conformément aux recommandations de la Commission Internationale de Protection Radiologique (CIPR) [2]. Afin de pouvoir répondre à ces obligations, les professionnels de la santé, médecins et paramédicaux, doivent être formés en radioprotection. En effet, la situation de la formation en radioprotection est préoccupante en Tunisie. Très peu d´études ont évalué les connaissances en radioprotection chez les travailleurs tunisiens [5-8]. Les résultats de l´étude de Kamoun *et al*. [5] reflètent un niveau faible chez les travailleurs exposés aux rayons X dans les salles opératoires d´orthopédie dans le grand Tunis (gouvernorats de l´Ariana, de Ben Arous, de la Manouba et de Tunis).

Dans l’étude de Marzouki [6] comparant les niveaux de connaissance en matière de radiation dans les services de radiologie, orthopédie et cardiologie, il a été démontré que le niveau de connaissance du personnel médical était globalement moyen dans les trois services. Le score des médecins radiologues était légèrement supérieur à celui de leurs confrères non radiologues. Cela pourrait être expliqué par les cours de radioprotection au programme du collège dédié aux résidents de radiologie au début de leur cursus et cela n´est pas le cas pour les autres spécialités. Aussi, une étude récente, publiée par Ben Hammamia *et al*. a montré que le score moyen des connaissances était à 8,15/20 chez le personnel d´une salle de cathétérisme endovasculaire à l´hôpital la Rabta à Tunis [8]. Dans notre étude, 50% des médecins ont eu un score de connaissance de moins de 50%. Bien qu´il n´existe pas de questionnaire standardisé pour l´évaluation des connaissances du personnel travaillant en rayons X, des études similaires faites dans des centres hospitaliers universitaires en Turquie [9], en Éthiopie [10] et au Cameroun [11] ont trouvé aussi un niveau de connaissances insuffisant.

Des limites de dose des RI ont été imposées assurant une protection appropriée aux personnes exposées. Selon les recommandations de la CIP [4], la limite de dose d´exposition annuelle corps entier pour les travailleurs est de 20msv/an, cette dose limite pouvant aller jusqu´à 50msv/an à condition de ne pas dépasser les 100msv sur 5 ans. A noter que 48,3% de notre population ne savaient pas la dose limite annuelle. Selon le rapport de la CIPR de 2012 [12], la dose maximale annuelle cumulée au cristallin a été abaissée de 150msv/an à une moyenne de 20msv/an sur 5 ans sans dépasser 50msv en une seule année. Le risque relatif de survenue d´une cataracte dans la population de cardiologues interventionnels par rapport au reste de la population varie de 2,6 à 3,3 selon Ciraj-Bjelac *et al*. [13]. Ceci nous incite à souligner dans nos résultats la méconnaissance de cet effet par 39,7 % des enquêtés et la surveillance ophtalmologique réalisée chez moins de 1 médecin sur cinq dans notre étude. Une surveillance dosimétrique spécifique du cristallin est proposée actuellement pour le personnel exposé aux rayons X notamment en radiologie interventionnelle [14]. Les nouvelles lunettes plombées apportent une atténuation d´au moins 90% du rayonnement incident et présentent un bon compromis poids- efficacité [15].

Dans notre travail, 60,3% des médecins n´ont jamais porté de dosimètre en raison de la non disponibilité (n=31), la perception de la non fiabilité des données dosimétriques (n=3) et l´oubli (n=1). La défaillance de la surveillance dosimétrique individuelle a également été observée dans une étude évaluant les connaissances en radioprotection des travailleurs au bloc d´orthopédie du Grand Tunis [5]. La panne du lecteur des dosimètres thermo luminescents au CNRP et la difficulté subie par l´établissement pour l´approvisionnement en nombre suffisant de dosimètres opérationnels sont les causes principales de la défaillance actuelle de la surveillance dosimétrique en Tunisie. Dans l’étude de la société d´angiographie cardiovasculaire et d´interventions (SCAI) ,76% ont déclaré porter leurs dosimètres, 8% ne le portait jamais, et 16% ont signalé qu’ils portaient leurs dosimètres de temps en temps [16].

Dans notre étude, la cache thyroïde, les lunettes en plomb et le calot en plomb ont été portés de façon constante chez 86,2%, 12,1%, 1,7% des médecins respectivement. Dans l’étude de la SCAI, l´utilisation par les enquêtés de la cache thyroïde, des lunettes plombées et de la protection pour les jambes était de 94%, 46%, 20% respectivement [16]. Jentzsch *et al*. ont montré que la compliance aux mesures de radioprotection (port de la cache thyroïde, dosimètre et blouse en plomb) était notée uniquement dans 54% des cas [17]. Des recommandations de radioprotection en cardiologie interventionnelle ont été publiées en 2013 par Duran *et al*. [18]. Aussi, Les recommandations de la CIPR [19] ont mentionné l´importance de l´enseignement de la radioprotection dans le cadre de la formation professionnelle initiale ou continue. Elle constitue en effet un pilier essentiel en termes d´assurance de la sécurité des travaux réalisés.

Dans notre travail, 57,14% des médecins ayant eu des grossesses alors qu’elles travaillaient en salle de cathétérisme et ont arrêté de travailler lors de la grossesse. Cela pourrait être expliqué par les connaissances insuffisantes et les mesures de surveillance inadéquates. Parmi celles qui ont continué de travailler en salle au cours de la grossesse, 66,66% ne savaient pas qu´elles étaient enceintes. Ainsi, 55,2% des médecins considèrent qu’une femme enceinte doit arrêter de travailler en salle de cathétérisme quel que soit sa position du travail. En effet, concernant les droits légaux des femmes enceintes travaillant en milieu médical et exposées aux RI, certains hôpitaux aux USA interdisent aux femmes de travailler près des rayonnements lorsqu’elles déclarent leur grossesse. Cette politique décourage les femmes employées de déclarer leur grossesse, ce qui protège l’institution de toute responsabilité des risques de l’exposition aux rayonnements car l’établissement n’a aucune responsabilité si la grossesse n’est pas déclarée [20]. Cependant, cela ne permet pas une surveillance adéquate de l’exposition aux rayonnements pendant la grossesse. En outre, les décisions judiciaires récentes ont interdit cette politique. Il existe une grande disparité dans l’approche de la travailleuse de la santé enceinte dans différents pays. En Espagne, un document de consensus spécifique sur la grossesse et la pratique hospitalière a été créé en 2002 au nom du Conseil espagnol de sécurité nucléaire et de la société espagnole de physique médicale. Selon la loi, le fœtus est considéré comme un membre du public, l’environnement des travailleuses enceintes doit garantir que le fœtus ne recevra pas plus de 1msv pendant toute la grossesse.

Malgré cela, ce travail a permis d´apprécier le niveau de connaissance et les pratiques adoptées dans la vraie vie, cependant la principale limite est le taux faible de participation au questionnaire. Il était inférieur à 50% dans notre enquête et il est jugé moyen comparé à d´autres études de même type [5,11]. Nous avons délivré un questionnaire anonyme. L´avantage de ce type de questionnaire est qu´il augmente le taux de participation. Cependant, l´inconvénient majeur est la surestimation du niveau de connaissance. En effet, le sujet enquêté peut consulter une référence sur internet ou un collègue.

## Conclusion

Cette étude montre que le niveau de connaissance et de pratique du Cardiologue Tunisien en matière de radioprotection est insuffisant. Ce qui devrait interpeller l´organisme tutelle dans ce domaine sur la nécessité urgente d´exiger une formation agréée qualifiante en radioprotection non seulement vis-à-vis du travailleur lui-même mais également du patient et de la population. Le cadre réglementaire tunisien de la radioprotection n´a pas changé depuis 1986, un guide de bonnes pratiques et un contrôle régulier des paramètres de radioprotection s´impose afin d´optimiser les procédures diagnostiques et interventionnelles et de prévenir les évènements indésirables.

### Etat des connaissances sur le sujet

- La cardiologie interventionnelle est un domaine d´exposition très élevé et du patient et de l´opérateur aux rayons X en médecine, classée en 2^e^ position après la radiothérapie;
- Les cardiologues ont un sur-risque de développer des cancers en particuliers celui du cerveau et des cataractes, par rapport à la population générale;
- Les procédures de cardiologie interventionnelle sont devenues de plus en plus complexes, avec une prolongation des durées d´examens et une surexposition aux rayons X.

### Contribution de notre étude à la connaissance

- Le niveau de connaissances des cardiologues en Tunisie dans le domaine de radioprotection est insuffisant;
- Il n´existe aucune corrélation entre le niveau de connaissances et de pratiques et la durée de l´expérience de l´opérateur;
- Les cardiologues interventionnels ont un niveau de connaissance et de pratique meilleur que les rythmologues et les cardiopédiatres.
